# An All-in-One Tool for 2D Atherosclerotic Disease Assessment and 3D Coronary Artery Reconstruction

**DOI:** 10.3390/jcdd10030130

**Published:** 2023-03-19

**Authors:** Savvas Kyriakidis, George Rigas, Vassiliki Kigka, Dimitris Zaridis, Georgia Karanasiou, Panagiota Tsompou, Gianna Karanasiou, Lampros Lakkas, Sotirios Nikopoulos, Katerina K. Naka, Lampros K. Michalis, Dimitrios I. Fotiadis, Antonis I. Sakellarios

**Affiliations:** 1 Department of Biomedical Research, Institute of Molecular Biology and Biotechnology—FORTH, University Campus of Ioannina, GR45110 Ioannina, Greece; 2 Unit of Medical Technology and Intelligent Information Systems, Department of Materials Science and Engineering, University of Ioannina, GR45110 Ioannina, Greece; 3 Department of Cardiology, Medical School, University of Ioannina, GR45110 Ioannina, Greece

**Keywords:** coronary artery disease (CAD), 3D reconstruction, OCT analysis, IVUS analysis, CTCA

## Abstract

Diagnosis of coronary artery disease is mainly based on invasive imaging modalities such as X-ray angiography, intravascular ultrasound (IVUS) and optical coherence tomography (OCT). Computed tomography coronary angiography (CTCA) is also used as a non-invasive imaging alternative. In this work, we present a novel and unique tool for 3D coronary artery reconstruction and plaque characterization using the abovementioned imaging modalities or their combination. In particular, image processing and deep learning algorithms were employed and validated for the lumen and adventitia borders and plaque characterization at the IVUS and OCT frames. Strut detection is also achieved from the OCT images. Quantitative analysis of the X-ray angiography enables the 3D reconstruction of the lumen geometry and arterial centerline extraction. The fusion of the generated centerline with the results of the OCT or IVUS analysis enables hybrid coronary artery 3D reconstruction, including the plaques and the stent geometry. CTCA image processing using a 3D level set approach allows the reconstruction of the coronary arterial tree, the calcified and non-calcified plaques as well as the detection of the stent location. The modules of the tool were evaluated for efficiency with over 90% agreement of the 3D models with the manual annotations, while a usability assessment using external evaluators demonstrated high usability resulting in a mean System Usability Scale (SUS) score equal to 0.89, classifying the tool as “excellent”.

## 1. Introduction

During the past decades, several efforts have been made to support clinicians in the evaluation of atherosclerosis in coronary arteries. These approaches involve the automatic or semi-automatic extraction of arterial geometry and atherosclerotic plaques from a variety of imaging modalities. The established coronary imaging techniques can be invasive and non-invasive. Invasive modalities include coronary angiography (CA), intravascular ultrasound (IVUS), and Optical Coherence Tomography (OCT), while non-invasive imaging of the coronary arterial tree can be achieved through Computed Tomography Coronary Angiography (CTCA).

Based on the utilization of these imaging modalities, or their fusion, a plethora of algorithms has been developed to enable the detection and 3D reconstruction of the coronary arteries and characterization of the atherosclerotic plaque types. These reconstructed geometries can then be used to support the evaluation of the disease by clinical experts and for the execution of in silico simulations, e.g., blood flow simulations, plaque growth, etc. [1]. 

Moreover, several commercial platforms and software are available currently for the analysis of coronary imaging. Such software is provided by Pie Medical Imaging, Netherlands, including the CAAS workstation which, among other things, allows CA analysis and the 2D analysis of OCT/IVUS, without, however, information about plaque composition or the 3D reconstruction of the vessels. Moreover, the 3mensio Coronary software enables the CTCA analysis. MEDIS Medical Imaging, Netherlands, also offers discrete solutions for the analysis of the most imaging modalities (Medis Suite XA, Medis Suite CT and the Medis Suite Intravascular). Finally, the major industry in coronary imaging, e.g., PHILIPS Healthcare, General Electric, etc., provides software and platforms which support their imaging vendors. 

In this work, for the first time in the current literature, we combine different methodologies in an all-in-one software which enables clinicians to assess coronary artery disease using either invasive imaging or non-invasive imaging. Through the developed and validated methodologies, semi-automatic reconstruction of the coronary arteries and plaques is achieved and quantitative assessment of the disease severity is provided. In particular, using the 3D reconstruction and plaque characterization tool, IVUS and OCT image processing analysis is performed, characterizing the lumen, outer wall borders, and plaque (struts can also be detected from the OCT cases). The quantitative coronary angiography (QCA) module provides the quantitative analysis of X-ray invasive coronary angiography (CA) enabling the reconstruction of segments or bifurcations. Moreover, the CTCA module enables the reconstruction of coronary trees and plaques, as well as the detection of stented regions. The tool allows the fusion of the results from the modules and hybrid reconstruction options (OCT/IVUS with the QCA or CTCA). To our knowledge, the tool is the only one available which enables comprehensive coronary imaging from various modalities. It is the first one which also combines the plaque characterization and their 3D visualization, as well as strut detection and the stent 3D reconstruction. The algorithms employed in the tool have been validated and the tool has been evaluated for its performance as well as for its usability.

## 2. Materials and Methods

### 2.1. Architecture

**Figure 1:**
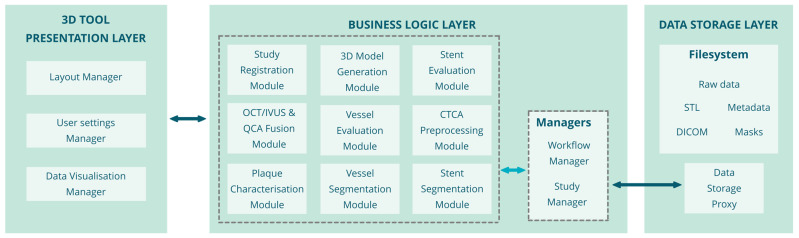
Architecture of the 3D coronary artery reconstruction and plaque characterization tool.

The overall architecture of the 3D reconstruction and plaque characterization tool is presented in Figure 1.

The 3D Reconstruction and plaque characterization tool is based on a three-tier architecture consisting of the following layers: (i) Data Storage layer: this layer contains the data of the patient to be analyzed and the filesystem where the data are stored. The data storage proxy handles the access to the database and the filesystem. The data stored in the filesystem are: the DICOM images of the patient to be processed, the segmentation masks as outputs of the reconstruction tool, the vessel and stent evaluation metrics and other metadata, and the 3D geometries of the vessel lumen, wall, plaques, and stent. (ii) Business Logic layer: This layer includes the modules that perform the image processing/analysis and create the 3D geometries of the vessel, plaques, and scaffolds. Those modules also extract measurements related to the lesion and the stent positioning. The user communicates with this layer through an API Gateway, selects the patient folder, and the Data Manager sends the corresponding data to the layer (back-end) modules to initiate the process. (iii) Presentation layer: includes the modules related to the user interface, the visualization of the data and the results and the configuration of the user interfaces (UI) and the algorithms. 

### 2.2. Modules

This section contains the description of the 3D Reconstruction and plaque characterization tool modules, shown in Figure 1, their functionality, and their input–output.

Workflow Manager: The functionality of each module depends largely on the type of image. The workflow manager is responsible for selecting the appropriate processing workflow for the imaging modality which is used.

Study Manager: This manager is the interface between the user and the Data Storage Proxy, where the user can select to view, edit, and process the specific patient data. In particular, when a DICOM image is analyzed from any imaging module, a patient’s “study” folder is created which includes all the produced results and metrics. 

CTCA Preprocessing Module: This preprocessing module applies a vessel enhancement filter in the DICOM images to remove irrelevant details and identify vessel candidate regions. This module is used in the case when CTCA images are processed.

- Input: CT DICOM images.
- Output: processed CT DICOM images using the Vesselness filter [2].

Vessel Segmentation Module: Segmentation of the vessel lumen, wall, and plaques. The segmentation method which is performed depends on the imaging modality.

- Input: The user inputs depend on the imaging modality (CTCA and CA case: starting and ending points of segment, OCT/IVUS case: lumen and adventitia borders), imaging data in DICOM format.
- Output: OCT, IVUS, CTCA: Vessel lumen and wall, calcified and non-calcified plaque segmentation masks; CA: Lumen and centerline path.

Vessel Evaluation Module: This module uses the outputs of the Vessel Segmentation Module, more specifically the segmentation masks and the extracted centerline paths, and provides several related metrics for the evaluation of the lesion.

- Input: Segmentation masks of the lumen, adventitia and plaques, 3D centerline path.
- Output: Lumen area and perimeter, outer wall area and perimeter, plaque burden.

Plaque Characterization Module: Plaque characterization enables the plaque components detection on IVUS, OCT, and CTCA images. The applied methodology depends on the imaging modality.

- Input: The required input is the masks of the lumen and outer wall borders.
- Output: OCT, IVUS: plaque segmentation masks, 3D point clouds, 3D surfaces of calcified plaques, metrics of plaque area, angle; CTCA: calcified and non-calcified plaques segmentation masks, 3D objects, volumes, areas of the calcified and non-calcified plaques.

3D Model Generation Module: This module uses as input the segmentation masks of the lumen, wall, and plaques, the 3D centerline path and the transformed 3D point cloud of the stent struts and performs triangulation and extra processing (clipping, remeshing) in order to create the 3D geometries of the vessel (lumen and outer wall), the plaques and the stent.

- Input: Segmentation masks of the lumen, adventitia and plaques, 3D centerline path, 3D stent transformed point cloud.
- Output: 3D models of the vessel, plaques and stent.

Study Registration Module: This module is used to co-register a study between the pre- and post-stenting phases of the operation.

- Input: 3D centerline path, 3D geometries of the vessel, plaques, and stent.
- Output: Co-registered 3D models.

The Stent Segmentation, Stent Fitting, Stent Evaluation and Centerline Projection Modules are employed only for OCT images. Moreover, for the Stent Fitting and Centerline Projection Modules, X-ray angiography images of the same study and a deformable model of the stent are available.

Stent Segmentation Module: This module performs automated stent strut detection from OCT and/or IVUS images.

- Input: Imaging data (DICOM series of the OCT/IVUS pullback), user input (manual annotations-corrections of detected stent struts).
- Output: Strut point cloud, metrics of struts (number of struts, malapposed struts, in-stent restenosis).

OCT/IVUS + Angiography Fusion Module: This module uses the 3D centerline path extracted from X-ray angiography images to transform the struts from the 2D frames to the original 3D space of the vessel. It is also used to transform the lumen, wall, and plaques into the 3D space.

- Input: 3D centerline points, segmented borders of the lumen and wall, strut point cloud.
- Output: Vessel lumen, wall, plaque lesions and strut point cloud in 3D space.

Stent Evaluation Module: Uses as input the 3D stent fitted model and the detected struts point cloud, and outputs several stent evaluation metrics to provide a series of outputs described below:

- Input: Strut point cloud, reconstructed 3D stent model.
- Output: Stent evaluation metrics (Stent CSA, minimum/maximum stent diameter, strut malapposition distance, % of unapposed struts, restenosis burden, fracture detection)

Data Visualization Manager: This module is used to display the segmentation results and related information to the user including 3D volume rendering, 3D vessel and stent models rendering, 2D classification maps of the segmentation, etc. It is also responsible for displaying the DICOM images and the calculated metrics of each component.

- Input: DICOM images, 3D models of the vessel, plaques and stent Vessel/plaque/stent evaluation metrics.
- Output: 3D rendering, 2D plots, visualization of the data.

User Settings Manager: This module enables the expert user to adjust the parameters of the various methods and filters, which are used to improve the results (e.g., DICOM window level, vesselness filter parameters, etc.).

Layout Manager: responsible for changing the layout of the User Interface in accordance with the used imaging modality and the type of required visualization.

### 2.3. User Interfaces 

The Graphical User Interface (GUI) of the 3D Reconstruction and Plaque Characterization tool consists of two windows visible simultaneously. The main window incorporates the menus for initiating or opening a case study of a specific patient and the parameters of the various segmentation algorithms. However, the main functionality of this window is the 3D Renderer where the reconstructed arteries are visualized to the user. The second window is the Image Viewer screen, where the layout changes form according to the loaded imaging modality. The Image Viewer screen has three types of layouts (IVUS-OCT, CTCA, QCA). The segmentation results and various metrics are displayed to the user on top of the original images in the Image Viewer. In addition, there are various functionalities allowing the user to manually improve the final segmentation results (border correction, calibration, etc.). 

The tool has been developed in C#, combined with Microsoft .Net Framework 4.6. Therefore, it is compatible with workstations running Microsoft Windows 7 or more recent versions. For the internal functionalities of the software (reading DICOM files, image processing, calculation of metrics, 3D rendering, post-processing of the reconstructed geometries) various third party libraries were used. DCMTK [3] and ITK [4] were used to read the DICOM images and the metadata required for the 3D reconstruction and to apply pre-processing filtering. EmguCV [5], a .NET wrapper for the OpenCV library, was used to extract segmentation metrics from the images. OpenCV [6] was used to create the segmented maps. VTK [7] was used to develop the 3D renderer module and VMTK [8] to calculate several metrics related to the arterial geometry (extract arterial segments, centerlines of the segments, length, degree of stenosis, etc.). The IVUS segmentation algorithm and the plaque characterization models were developed using the Python language and the Keras [9] deep learning library. The OCT segmentation algorithm (lumen, adventitia, stent detection), the 3D fusion module, the QCA module, and the CTCA segmentation algorithm were all developed using the C++ language. 

### 2.4. Algorithms

#### OCT Analysis

Analysis of OCT images provides the lumen and outer wall borders, the plaque components, and the struts of the stent. The first step is related to the lumen and outer wall border segmentation, which starts with guidewire artifact removal. This is achieved using the Harris–Stephens detector scheme. Bilateral filtering is employed to smooth out any residual noise and enhance the lumen border. Then, for the lumen extraction, a Fast Marching algorithm is applied. In order to initialize the algorithm, we need to define a speed function on which the Fast Marching algorithm operates and set the starting and ending seed points of the shortest path. A novel algorithm for the segmentation of the outer border of the adventitia layer was also developed. The methodology is based on the detection of the sharp transition effect between bright areas representing the tissue to dark areas representing background pixels. Initially, we perform bilateral filtering of the OCT frame. Next, a standard deviation filtering is performed on each image for a 11 × 11 neighborhood. The value of each output pixel is the standard deviation of the 11 × 11 neighborhood around the corresponding input pixel. The final step of the methodology is to apply the Frangi vesselness filter [2] on the image of the standard deviation, to find the outer border of the adventitia. This is required because a healthy media tissue (the pixels of the dark ring in the figure of the standard deviation) has imaging features like a vessel (tubular and continuous structure). Therefore, using a Frangi vesselness filter with a thickness parameter of 31 pixels provides us with the final adventitia border. 

For the characterization of the different plaque types, a new methodology was developed, which is based on deep learning techniques. Specifically, a convolutional neural network (CNN) was trained to identify four different types of plaque from OCT images (calcified plaque (CA), fibrous tissue (FT), lipid tissue (LT), mixed tissue (MT)). The network architecture contains nine convolutional (CONV) blocks and two fully connected (FC) layers. Each CONV block is a sequence of layers and consists of a 2D CONV Layer, a Batch Normalization Layer and a Rectified Linear Unit layer. Two max pooling layers were placed after the third and the sixth convolutional blocks, respectively. One average pooling layer was placed after the ninth convolutional block. The spatial support of the filters in each of the convolutional layers was set to 3 × 3 pixels. The number of the filters in the first three CONV layers was set to 32. In order to compensate for the information loss caused by max pooling, the number of filters in second CONV block and third CONV block were set to 64 and 128, respectively. Two FC layers followed the global pooling layer. The first FC layer included 512 neurons and the second one included three neurons. One dropout was set between these two FC layers with a dropout ratio of 0.5 to further avoid overfitting. Finally, a Softmax layer was placed at the end of our classifier to produce probability scores and the pattern is classified to the class with the highest output probability. The plaque characterization step is executed right after the extraction of the lumen and adventitia borders. This is crucial, because the methodology needs the borders to define the region of interest (ROI) of the tissue where the neural network is trained and tested. For the sake of completeness, it should be pointed out that the CNN generates predictions for five classes: four classes representing the four plaque types (CA, FT, LT, MT) and one class [background (BK)] representing the rest of the pixels, e.g., catheter shadow pixels, healthy tissue pixels. Two different experts discriminated four types of plaque on 400 OCT frames. From the 400 annotated frames, 300 frames were used for training and 100 frames were kept apart to be used for testing. Furthermore, from the 300 training frames, 1000 patches (pixels) were randomly selected from each frame, using the procedure described previously. Each patch was augmented, by rotating it 90 and 180 degrees, to enhance the generalization of the model, thus resulting in a dataset of 900,000 samples. Finally, a 70–30 split was used to split the dataset into training and validation sets. Therefore, the dataset contains 630,000 training patterns, 270,000 validation patterns, and 100 frames were used solely for the testing. The patches included in the validation set did not come from the same OCT frames as some of the patches in the training set. 

For the detection of metallic stent struts, we extended the method of Wang et al. [10] by including a clustering algorithm to cluster each detected strut in a separate cluster in order to extract stent apposition metrics. Specifically, the first step of the strut detection algorithm is to clear out the polar frame from any residual noise and to enhance the shadowed regions. To achieve this, percentile thresholding is used. Next, we locate the candidate strut pixels in the polar frame. The main criteria to classify if a scan line contains a strut candidate is its shadow length and its slope. The parameters used here for the classification of candidate strut pixels are the slope of the brightness profile and the length of the shadowed regions. Next, the detected candidate struts are clustered in a separate cluster, using the DBSCAN algorithm [11]. Besides the metal struts, the tool is capable to detect bioabsorbable vascular stent (BVS) struts. The BVS struts have a rectangular morphology where the borders of the rectangle are brighter than the core of the strut, which is usually dark. For this purpose, the OCT images are first converted to grayscale and then they are binarized, using a threshold of 0.15. In addition, a set of morphological operations is performed, with the first being a morphological closing of the image using a disk with one-pixel radius and the second being a filter to fill the holes of the image. Subtracting the thresholded image from the image with the filled holes gives us the possible strut locations. In order to clear any false positives, a set of morphological rules is applied, using a priori information about the BVS struts’ morphology. More specifically, for the final result, only structures with an area between 10 and 40 pixels and aspect ratio < 5 are selected [12]. Finally, the BVS struts should be located within a 10-pixel width band around the lumen border. 

### 2.5. IVUS Analysis

A total of 4,197 annotated IVUS frames were used for the training of a U-net-based deep learning model to segment the lumen and adventitia boundaries of the artery. The aforementioned model includes the “Atrous Spatial Pooling layers” and “squeeze and excitation” blocks which boost the architecture to extract more meaningful features. 

IVUS images can be used for the characterization of the various plaque types present in the vessel tissue. For this purpose, a methodology based on deep learning techniques was developed, as in the OCT case. Because the methodology for IVUS images is based on the one developed for OCT, in this section we focus on the changes made to incorporate the OCT model for IVUS plaque characterization and present the validation strategy and results. One expert discriminated four types of plaque (Calcified Plaque, Fibrous Tissue, Lipid Tissue, Fibro-fatty tissue) on 380 IVUS frames. From the 380 annotated frames, 295 frames were used for training and 95 frames were kept apart to be used for testing. Furthermore, from the 295 training frames, 1000 patches (pixels) were randomly selected from each frame, with each patch having a size of 25 by 25 pixels. The IVUS patch is smaller than the OCT, because of the lower resolution of the IVUS images. Each patch was augmented by rotating it 90 and 180 degrees to enhance generalization of the model, thus resulting in a dataset of 900,000 samples. Finally, a 70–30 split was used to split the dataset into training and validation sets. Therefore, the dataset contained 60,000 training patterns, 265,000 validation patterns, and 95 frames used solely for testing. The model used for the plaque characterization is a Convolutional Neural Network. 

The main changes from the OCT architecture can be found in the number of filters of the Convolutional Layers of the nine convolution blocks. The number of the filters in the first three CONV layers was set to 16, while the number of filters in the second convolution block and third convolution block were set to 32 and 128, respectively. 

### 2.6. QCA Analysis

The developed methodology is based on a previously developed approach [13]. However, it has been significantly improved, since the current methodology provides the 3D reconstruction of coronary bifurcations, a methodology that is validated with satisfactory results [14]. The proposed approach consists of three steps. Initially, the 2D lumen borders and centerlines are detected. Then, the 3D bifurcation path is extracted and the 3D lumen borders are reconstructed around the 3D bifurcation path and finally, the main and side segments are combined in order to produce the final model of the bifurcated artery. 

### 2.7. CTCA Analysis

The current literature contains several studies which present the segmentation and analysis of CTCA images [15,16,17]. In this work, the 3D reconstruction and plaque characterization tool integrates a methodology previously developed and validated in [18,19,20]. Briefly, the methodology consists of six different steps and is implemented with 2D axial CTCA images. The proposed technique provides a detailed geometry for the lumen, the outer wall, the calcified plaques (CP), and noncalcified plaques (NCP). In the first step, a preprocessing Frangi vesselness filter is applied for an initial identification of vessel structures and then a coronary vessel centerline extraction methodology is applied, based on a minimum cost path based approach. Subsequently, weight functions are estimated for the lumen, the outer wall, and the CP. For the lumen, a combination of two sigmoidal membership functions are utilized, whereas for the outer wall and the CP two sigmoidal functions are utilized for the segmentation procedure. The idea of weight function estimation is that its component weight function poses a possibility of its voxel to be characterized as lumen, outer wall, or CP voxel. The weight functions are adapted around specific threshold values for the lumen, the outer wall, and the CP, which are defined by the user through a calibration procedure. More specifically, the user manually annotates some voxels, which correspond to the lumen, around the region and the coronary stent, and additionally voxels of the outer wall, the CP, and the NCP are annotated. This calibration procedure leads to the estimation of the lumen, outer wall, and CP weight functions, which is utilized for the segmentation procedure. Subsequently, a 3D level set-based segmentation technique is implemented for the segmentation of the lumen, the outer wall, and the CP. As far as the coronary stent detection is concerned, the identification of the voxels which correspond to the coronary stent region is based on a procedure similar to the aforementioned one. More specifically, a sigmoidal membership function was defined for the identification of coronary stent position and length, which is fully adapted to the mean luminal intensity. 

## 3. Results

Validation of the OCT borders segmentation algorithm demonstrated that the algorithm is accurate enough to detect and segment the borders of lumen (R = 0.99) and outer wall (R = 0.77). The average Hausdorff Distance (HD) was 0.097 mm and the Dice Similarity was 0.96 [21]. Moreover, the validation results of the proposed plaque characterization algorithm demonstrated overall accuracy of 85.6%. The struts detection algorithm presented a regression coefficient of 0.82 between the manual annotations and the automatic detection of the stent struts.

The deep learning methodology for IVUS segmentation is characterized by the increased generalizability of the model due to the utilization of data acquired by different IVUS systems (Philips VOLCANO, Boston Scientific) and this resulted in a Dice coefficient of 89% and 90% for lumen and adventitia borders, respectively, while the Jaccard index was 81% for both borders. The plaque characterization methodology had overall accuracy 91.43%.

Finally, the validation of the QCA-based artery segmentation and reconstruction demonstrates that the generated models have a high resemblance to those annotated by the expert 2D lumen borders. In detail, considering the dataset of the 26 coronary bifurcated arteries, the mean HD between the 2D borders of the model and the 2D annotated lumen borders is 0.34 mm for the first angiographic view and 0.26 mm for the second one. The dataset’s mean similarity between the recovered 2D lumen borders of the model and the true annotated 2D lumen borders is 0.93 for both the X-ray angiographic views. The mean similarity is >90% and in some cases, the similarity is 99%.

### 3.1. D Fusion Evaluation

**Figure 2:**
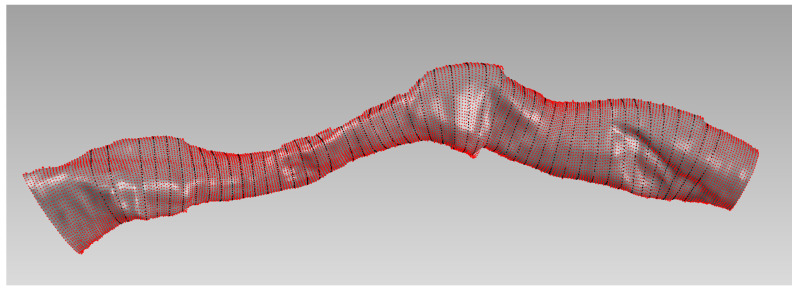
Case example for the evaluation analysis. The red points represent all the OCT-detected borders used for the reconstruction. The black points represent the cross-sections of the 3D model, which is presented as the grey surface.

The 3D artery reconstruction and plaque characterization tool enables the generation of arterial geometries based on the fusion of IVUS/OCT imaging with the centerline extracted by the QCA (Supplementary Materials). The evaluation of the 3D fusion algorithm, and specifically, the 3D reconstructed arteries, are compared to the 2D lumen borders detected on OCT frames. Comparison was performed for the lumen area and diameter. Each reconstructed artery is divided into 0.5 mm cross-sections, which are registered with the OCT frames. A case example is shown in Figure 2. 

**Figure 3:**
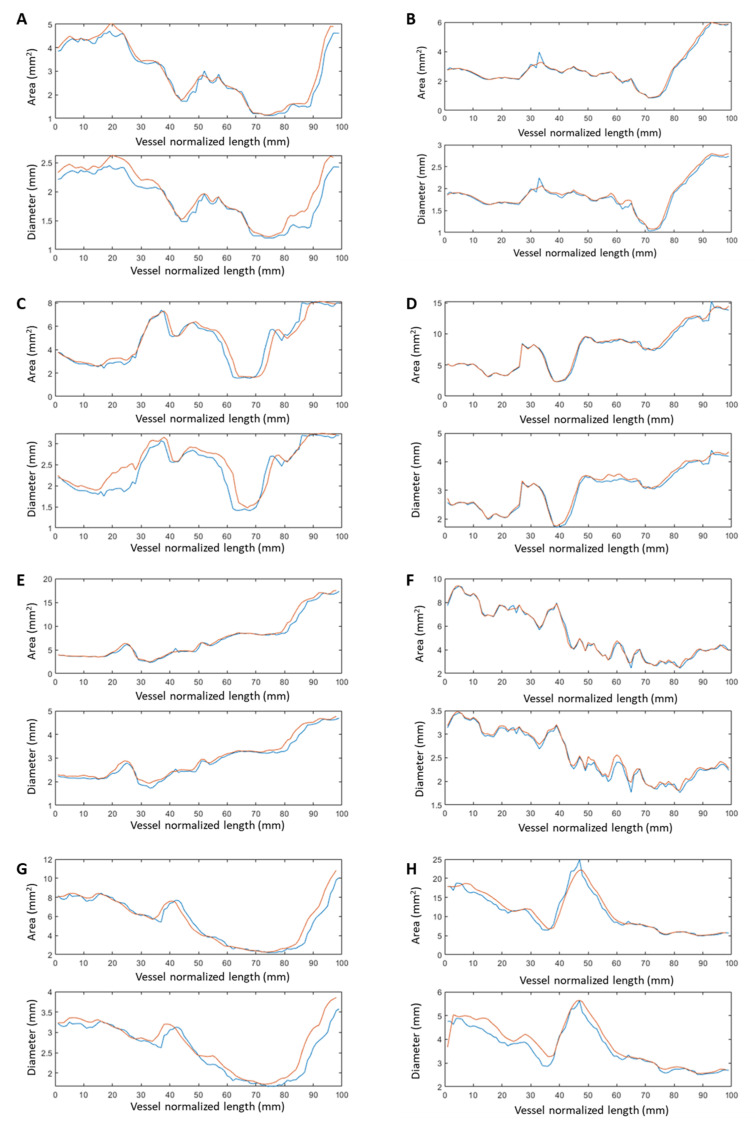
Comparison of area and diameter between the 3D model cross-sections (orange line) and the 2D OCT frames (blue line) for the number of patients we processed. The cases (**A**–**H**), represent the analysis of the respective eight patients.

However, the number of OCT frames are significantly more than the 3D model’s cross-sections. For this reason, the registration was based on landmarks visible on OCT frames (stent, bifurcations) and the 3D model (the bifurcations were artificially created to be present on the point cloud of the 3D artery). As metrics of comparison, the correlation coefficient, the least residual sum of squares error, and the Bland–Altman plots were selected. Experts performed the 3D reconstruction of the arterial segment and the registration was achieved with the supervision of an experienced cardiologist. Data from eight patients were used for this analysis and the comparison is presented in Figure 3. 

**Figure 4:**
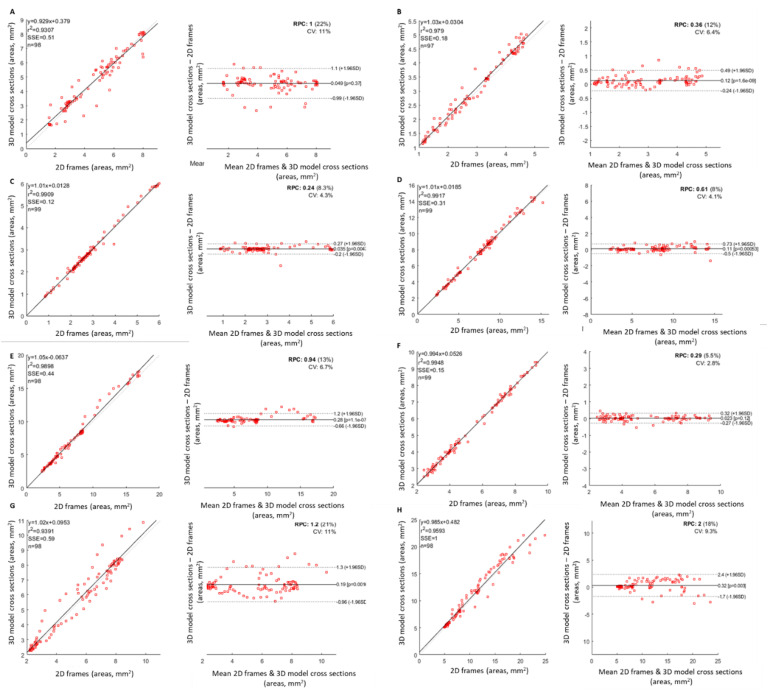
Regression and Bland–Altman plots for the lumen area for the number of patients we have processed. The cases (**A**–**H**) represent the analysis of the respective eight patients.

The evaluation analyses demonstrated a good agreement between the 3D models with the findings from the 2D analysis. In particular, it was found that regarding the lumen area, the correlation coefficient r^2^ between the 3D cross sections and the 2D borders is 0.97. The correlation coefficient r^2^ for the lumen diameter is 0.95. The minor difference is due to the smoothing operation during the generation of the triangulated surface of the 3D model, and in addition due to the minor difference during the registration of the 3D model with the 2D frames, which is clear in the vessel curvilinear length graphs. The overall regression and Bland-Altman results for the eight patients are presented in Figure 4. 

### 3.2. Usability Assessment

The 3D reconstruction and plaque characterization tool has been developed within the framework of the InSilc project [22]. The usability assessment of the tool was performed using external evaluators. In particular, the tool was presented at four sites (Harvard Medical School, Boston, Massachusetts, USA; Medstar Washington Hospital, Washington DC, USA; University College of London, UK; University of Ioannina, Ioannina, Greece) with significant experience in analyzing coronary arterial imaging and performing 3D coronary arterial reconstructions. 

In order to perform the usability, a questionnaire was prepared and delivered to all users to be filled. The demographics of the evaluators are presented in Table 1.

The tool was tested by nine evaluators from the clinical (33% were cardiologists, 11% were interventional cardiologists) and research domains (56%). Of the users, 76% had expertise less than 5 years, 13% had 11–15 years of experience, and 13% with more than 13 years of experience. All evaluators had good computer skills. Most of the evaluators used computers everyday (75%), whereas some evaluators used computers all day (25%). 

The evaluators were asked to use the tool and provide their feedback on the complexity of the usage of single modules/components, as well as of the entire tool and the complexity of techniques involved. For the integrated 3D tool, the evaluators found the complexity very low (56%) and not complicated at all (44%). Regarding the IVUS module, 44% of the users found that the IVUS border segmentation and the plaque characterization modules were not complicated at all, while 56% found that the modules introduced a very limited or limited complexity. As far as the OCT module is concerned, most of the evaluators (89%), found the functionality of the borders segmentation not complicated, while 44–56% found that the plaque characterization and the stent detection functionalities are of very limited complexity. Among all the 3D modules, it seems that the one that is perceived to be more complex compared to the rest of the modules is the QCA, with 67% of the evaluators finding this module to have a very limited complexity. Concerning the CTCA module, 78% of the evaluators believed that there is no complexity for the border segmentation functionality, the plaque characterization, and the reconstruction functionalities.

The tool can be used by the different evaluators to support decision making for the management of CAD patients. All evaluators were asked to provide their feedback on this intended use. Again, the effectiveness was evaluated for each module separately and for the tool as a whole. In total, 78% of the evaluators found the effectiveness of the integrated tool fully satisfactory, while the rest of the evaluators (22%) replied that this effectiveness is partially satisfactory. According to the replies, the modules that seem to be more effective are the OCT and the CTCA. In more detail, 78% of the evaluators found that the OCT border detection and plaque characterization, the X-ray (3D QCA)-based reconstruction, the CTCA borders segmentation module and the integrated tool as a whole are fully satisfactory. The module with the least satisfied users is the IVUS borders segmentation module, with which only 56% of the evaluators were fully satisfied and the rest of the evaluators (44%) were partially satisfied. Finally, as far as the IVUS plaque characterization module, the CTCA plaque characterization module, and the 3D artery reconstruction based on fusion of IVUS/OCT with QCA are concerned, 89% of the evaluators voted that they are fully satisfied. 

Apart from the effectiveness of the integrated tool, the interface, the way the information is provided, and the use of language play a very important role on the acceptability of the proposed software solution. Of the evaluators, 78% (rating 9/10 and 10/10) found that the interfaces are very easy to use, 88% (rating 9/10 and 10/10) of the users found that the interfaces are easy to follow, and 78% (rating 9/10 and 10/10) that the notification messages are only presented at appropriate times. In addition, all evaluators voted that the menus are clear and unambiguous (rating 9/10 and 10/10). 

In addition, all evaluators agreed on the attractiveness of the interfaces, the satisfactory “look and feel” sensation and the easy and straightforward interconnection and navigation options (rating 8/10–10/10). Only 11% of the evaluators were not satisfied with how one screen works, however, even for these replies, the rating was far above the average (7/10). 

The next criteria for the evaluation of this software solution were the individual modules and the integrated tools performance. In more detail: (i) 67% of the evaluators found satisfactory the performance of the IVUS borders segmentation module, the OCT plaque characterization module, and the X-ray (3D QCA)-based reconstruction, (ii) 78% of the evaluators found satisfactory the performance of the IVUS plaque characterization module and the OCT-based stent detection, (iii) 89% of the evaluators found satisfactory the performance of the CTCA plaque characterization and reconstruction modules, the 3D artery reconstruction based on fusion of IVUS/OCT with QCA, and the tool as a whole. The remaining percentages of the evaluators voted for an acceptable performance for the aforementioned modules. Only for the OCT-based stent detection, there was a small percentage of evaluators (10%) who found the performance unacceptable. 

As far as the efficiency of the integrated tool and the individual modules are concerned, almost all evaluators believed in the provided effectiveness in the field of CAD research and on decision making (rating 7/10–10/10). However, 11% of the evaluators were not confident about how stent reconstruction could improve CAD management (rating 5/10). This was a general comment and not related to the usability of the tool. Regarding the overall intention of the users on using the 3D reconstruction and plaque characterization tool, it seems that 78% of the evaluators were interested in using the whole tool, while the rest of the evaluators (22%) were more interested in using only the IVUS and OCT module and the 3D reconstruction module.

**Figure 5:**
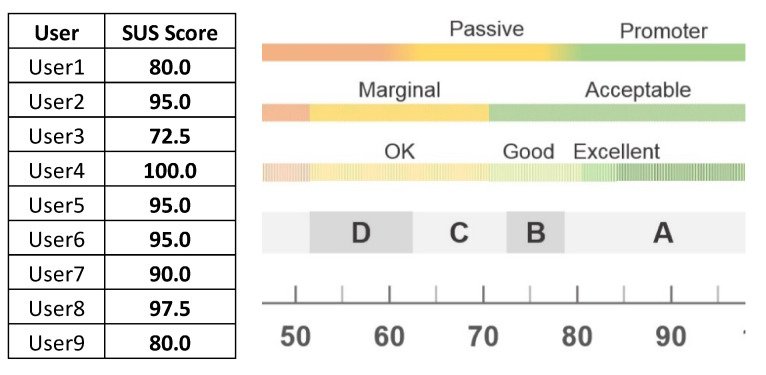
Results from SUS score [23].

In addition, the evaluators were asked to express their feelings after using the 3D coronary artery reconstruction and plaque characterization tool. In more detail, the System Usability Scale (SUS) [23] was used. In fact, the SUS score is not only intended to track the perceived ease-of-use, but in addition it can provide a global measure of system satisfaction and sub-scales of usability and learnability. The mean SUS score was 89.4 ± 9.0, classifying our tool as excellent according to the SUS scale (Figure 5). 

## 4. Discussion

The main contribution of this work in the current literature is the development of a novel and comprehensive all-in-one tool for the 3D reconstruction and plaque characterization which enables the reconstruction of the coronary arteries using several imaging modalities or using a fusion of them (IVUS or OCT and X-ray angiography). Furthermore, its modules include novelties in terms of implementation and outcomes. Accuracy of all algorithms is in most cases >90% which enables the accurate assessment of atherosclerotic disease. Manual correction is also provided as a functionality of the tool to further increase the resulting accuracy. For the first time, we combine the level set algorithm with clustering techniques for lumen segmentation. The developed methodology works well with both main IVUS modalities (Boston and Volcano), while in previous studies the methodologies were based only on one type of IVUS imaging (Boston or Volcano). Regarding OCT analysis, our developed methodology works independently of the stent presence. In case of stented artery, the struts are fully automatically detected and used for the 3D reconstruction of the stent scaffold. Additionally, it includes many novel characteristics for removing the catheter artefacts, an issue which has not been addressed in the previous methodologies (for example, in some OCT studies the catheter artefact is in touch with the lumen border). The strut detection methodology was applied to a large dataset of stented arteries with different stent types and the results demonstrate that the methodology for strut detection is independent of the stent type (bare metal stents and bioresorbable vascular scaffolds). Regarding the CTCA, our methodology is the only one which provides the scaffold interface with the lumen border. The results show that the scaffold can be accurately detected. Finally, regarding the QCA methodology, we have developed a semi-automated methodology for the reconstruction of coronary bifurcations. The minimum user interaction is that it requires to set the starting and ending points, while the lumen centerline and the lumen borders are extracted automatically. Overall, the 3D reconstruction and plaque characterization tool combines and includes several novel functionalities such as the automated calculation of significance for the disease assessment metrics or the manual correction of the identified borders in several views all simultaneously registered, and considering also the previous or afterwards analyzed frames to reduce the required analysis time and improve the usability.

Currently, there are many commercial software packages available which can be used for the clinical assessment of atherosclerotic disease. The main advantage of our developed tool is that it is an all-in-one tool which enables the analysis of the most used imaging modalities of coronary imaging, e.g., IVUS, OCT, X-ray angiography, and CTCA. Another major advantage over the available commercial software is that our tool provides fully automatically the reconstruction of the arterial models which can be used directly for simulation purposes. The reconstruction can be achieved using any 3D centerline generated by QCA or CTCA and the borders from IVUS or OCT. The CTCA-based reconstruction enables the analysis of the whole arterial tree. Moreover, the plaques as well as the stent for the first time are visualized in 3D space as objects. 

Another major advantage of the developed 3D reconstruction and plaque characterization tool is that all the results which are produced from any patient analysis are automatically extracted and stored. It enables 2D assessment of the atherosclerotic disease providing a plethora of metrics such as the lumen and outer wall areas and diameters, the plaque composition, and the degree of stenosis, as well as stent-related metrics such as the number of struts per frame, the in stent restenosis, the degree of malapposition and other. Thus, they can directly be used for further analysis either for clinical or research purposes. Moreover, the 3D reconstructed objects are extracted for simulation purposes such as the estimation of shear stress [24,25] or the non-invasive calculation of the fractional flow reserve [26]. Future steps in the development include further evaluating its performance by installing it into more clinical centers. Moreover, as performed previously with other tools of our lab, the reconstructed results will be used as input to other tools such as the SmartFFR calculation [27], the prediction of atherosclerotic plaque growth, or the simulation of stent deployment. 

The current tool has some limitations. The first limitation regards the 3D reconstruction based on the QCA approach. In that case, the tool assumes that there is at least 30 degrees of difference between the two angiographic views. In smaller angle differences, the results are questionable. Another limitation is that the development of the tool is based on the user requirement that has to be almost fully automatic. For this reason, it has been developed to read DICOM images with the tags included. Without the DICOM tag information, the tool is not functionable. Finally, the CTCA-based reconstruction has been developed on protocols established during the implementation of the presented software. Newer protocols using the last generation CT scanners may require some re-calibration of the algorithms.

## 5. Conclusions

We presented a single tool for the reconstruction of coronary arteries, the plaques and the stent integrating algorithms for the analysis of IVUS, OCT, CA and CTCA images. The tool is unique since it provides all the options for the analysis of the coronary network imaging. The final results are also visualized in 3D space, while the results are extracted in a format ready for further analysis for simulation purposes. The evaluation and usability assessment of the tool demonstrated an excellent performance and effectiveness to analyze and assess the coronary arteries.

## Figures and Tables

**Table 1:** Demographics of the evaluators.

Age	30% under 30 years old, 56% 30–39 years old, 11% over 40 years old
Experience	37% less than 2 years, 37% 2 to 5 years, 13% 6–10 years, 13% 11 to 15 years
Profession	44% Cardiologists/radiologists, 56% stent industry, researchers, CROs
Computer use	75% everyday, 25% all the time

## Data Availability

The data are not available due to ethical reasons.

## Supplementary Materials

The following supporting information can be downloaded at: https://www.mdpi.com/article/10.3390/jcdd10030130/s1, Figure S1. Case example of plaque characterization and borders segmentation using OCT imaging. The Show Annotations button enables the visualization of the plaques. Figure S2. Several metrics are provided for the detected struts. Figure S3. Case example of plaque characterization and borders segmentation of IVUS images. Figure S4. Segmentation of the 2D lumen borders using the QCA module. Figure S5. 3D reconstruction of a coronary bifurcation including the lumen (red), the outer wall (green), the calcified plaques (white) and non-calcified plaques (yellow) using CTCA imaging.
