# Supplementary material for: An All-in-One Tool for 2D Atherosclerotic Disease Assessment and 3D Coronary Artery Reconstruction
Source: J Cardiovasc Dev Dis. 2023 Mar 19;10(3):130. doi: 10.3390/jcdd10030130 (PMC10056488; doi:10.3390/jcdd10030130)
Supplement: Supplementary file 1 [file jcdd-10-00130-s001.zip › jcdd-2273644-supplementary.pdf]

Journal of
Cardiovascular Development and Disease

Supplementary Materials:

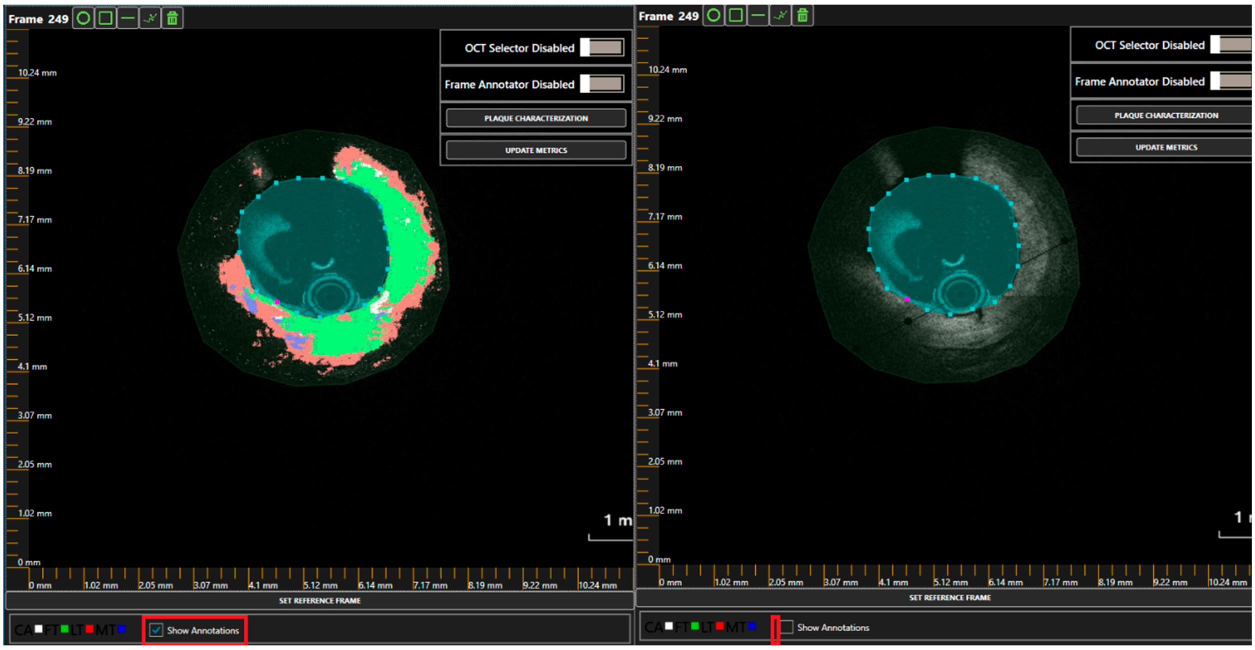

Figure S1. Case example of plaque characterization and borders segmentation using OCT imaging. The Show Annotations button enables the visualization of the plaques.

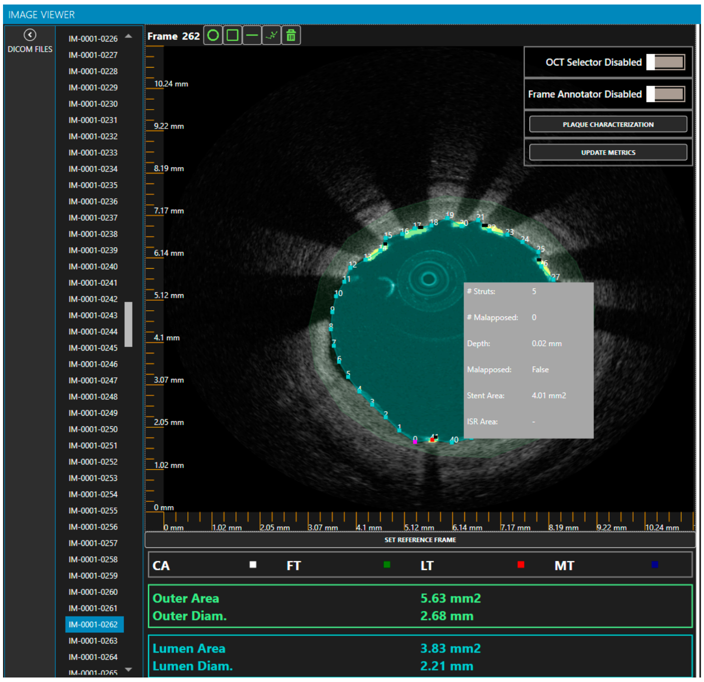

Figure S2. Several metrics are provided for the detected struts.

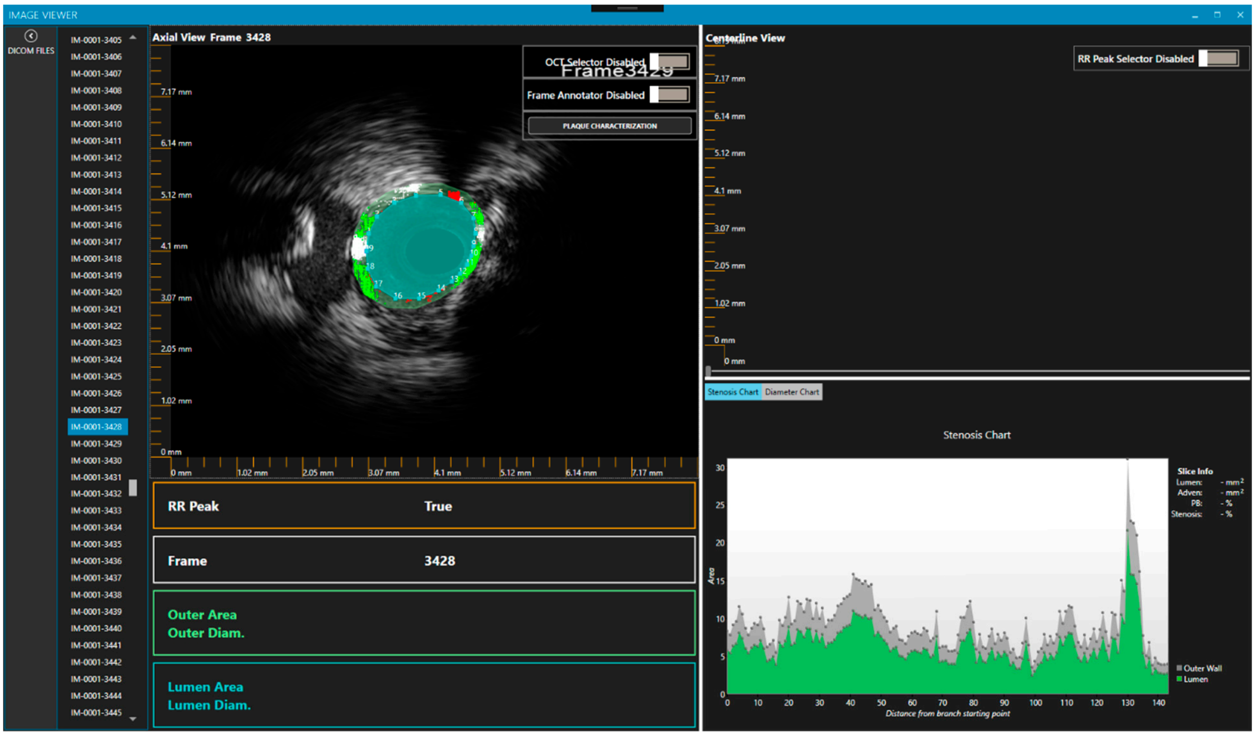

Figure S3. Case example of plaque characterization and borders segmentation of IVUS images.

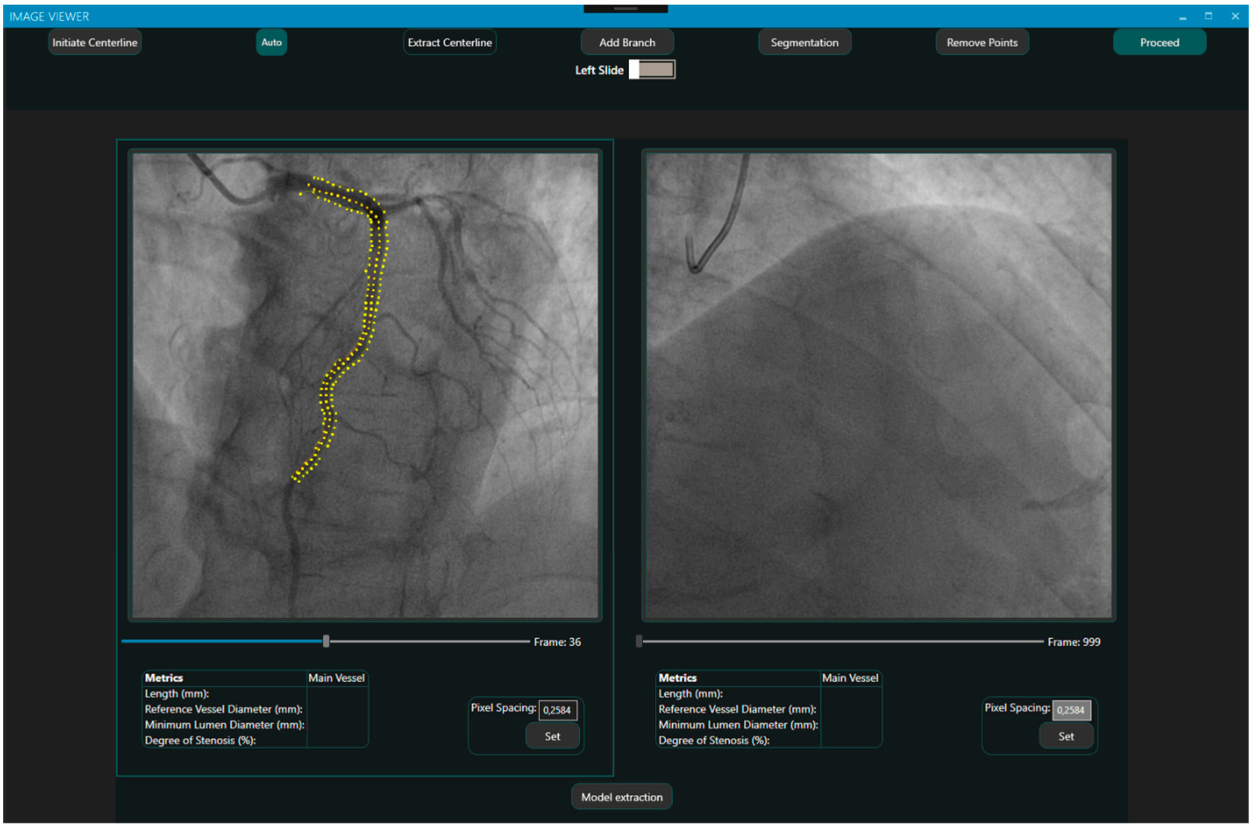

Figure S4. Segmentation of the 2D lumen borders using the QCA module.

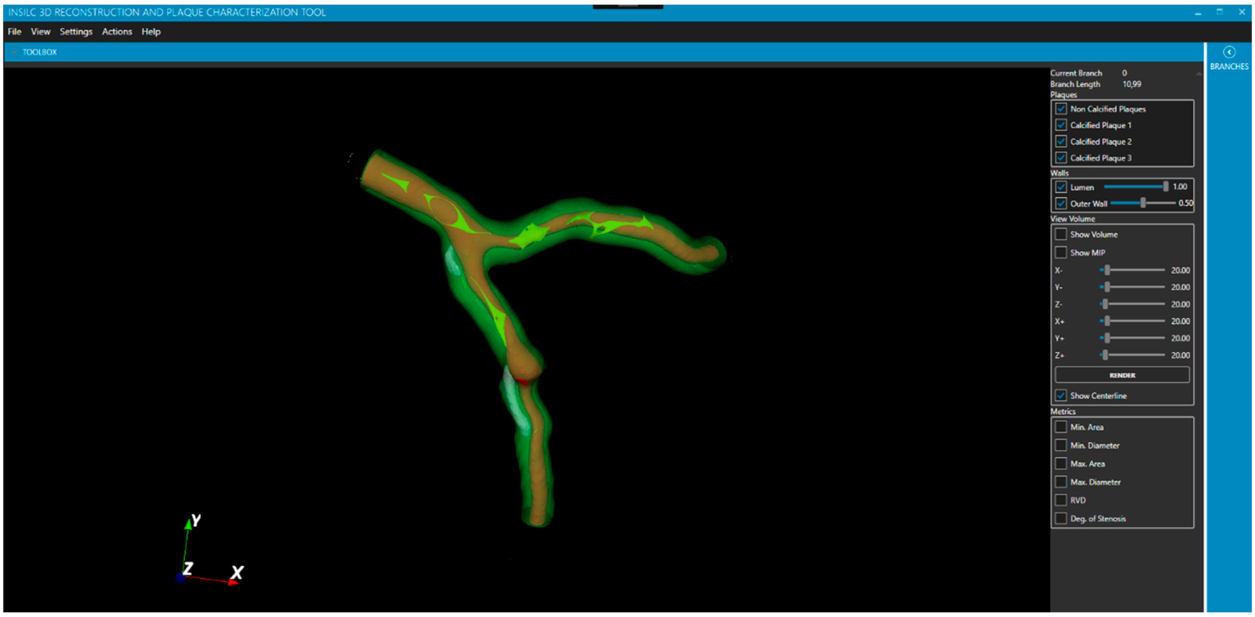

Figure S5. 3D reconstruction of a coronary bifurcation including the lumen (red), the outer wall (green), the calcified plaques (white) and non-calcified plaques (yellow) using CTCA imaging.
